# Neurotransmitter Specific, Cellular-Resolution Functional Brain Mapping Using Receptor Coated Nanoparticles: Assessment of the Possibility

**DOI:** 10.1371/journal.pone.0145852

**Published:** 2015-12-30

**Authors:** Ebrahim Forati, Abas Sabouni, Supriyo Ray, Brian Head, Christian Schoen, Dan Sievenpiper

**Affiliations:** 1 Electrical and Computer Engineering Department, University of California San Diego, La Jolla, CA 92098, United States of America; 2 Biomedical Sciences, University of California San Diego, La Jolla, CA 92098, United States of America; 3 Department of Anesthesiology, University of California San Diego, La Jolla, CA 92098, United States of America; 4 Nanopartz Inc., Loveland, CO 80537, United States of America; Argonne National Laboratory, UNITED STATES

## Abstract

Receptor coated resonant nanoparticles and quantum dots are proposed to provide a cellular-level resolution image of neural activities inside the brain. The functionalized nanoparticles and quantum dots in this approach will selectively bind to different neurotransmitters in the extra-synaptic regions of neurons. This allows us to detect neural activities in real time by monitoring the nanoparticles and quantum dots optically. Gold nanoparticles (GNPs) with two different geometries (sphere and rod) and quantum dots (QDs) with different sizes were studied along with three different neurotransmitters: dopamine, gamma-Aminobutyric acid (GABA), and glycine. The absorption/emission spectra of GNPs and QDs before and after binding of neurotransmitters and their corresponding receptors are reported. The results using QDs and nanorods with diameter 25*nm* and aspect rations larger than three were promising for the development of the proposed functional brain mapping approach.

## Introduction

Chemical messengers released by neurons, known as neurotransmitters, enable nerve fibers to communicate signals to the other nerves in their vicinity. Since various aspects of brain circuitry are modulated by neurotransmitters [1], an understanding of neurotransmitter’s dynamics helps to comprehend brain functions. This is difficult to study due to their small size and low concentration. So far scientists have identified more than 100 neurotransmitters and more are yet to be discovered. Dopaminergic neurons, which use dopamine as a neurotransmitter for neural signaling [2], are of special importance as defects in this type of neural signaling have been associated with various disorders such as Parkinson’s disease [3], Alzheimer’s disease [4], and schizophrenia [5–8].

In recent years investigators have developed a number of methods to detect acute changes in neurotransmission [9]. Most of these methods were based on molecular imaging techniques, in which changes in receptor kinetic parameters were detected by administering a radio-labeled ligand and measuring its concentration by Positron Emission Tomography (PET), Single Photon Emission Computed Tomography (SPECT) camera, or Functional MRI (FMRI) [10–15].

**Fig 1:**
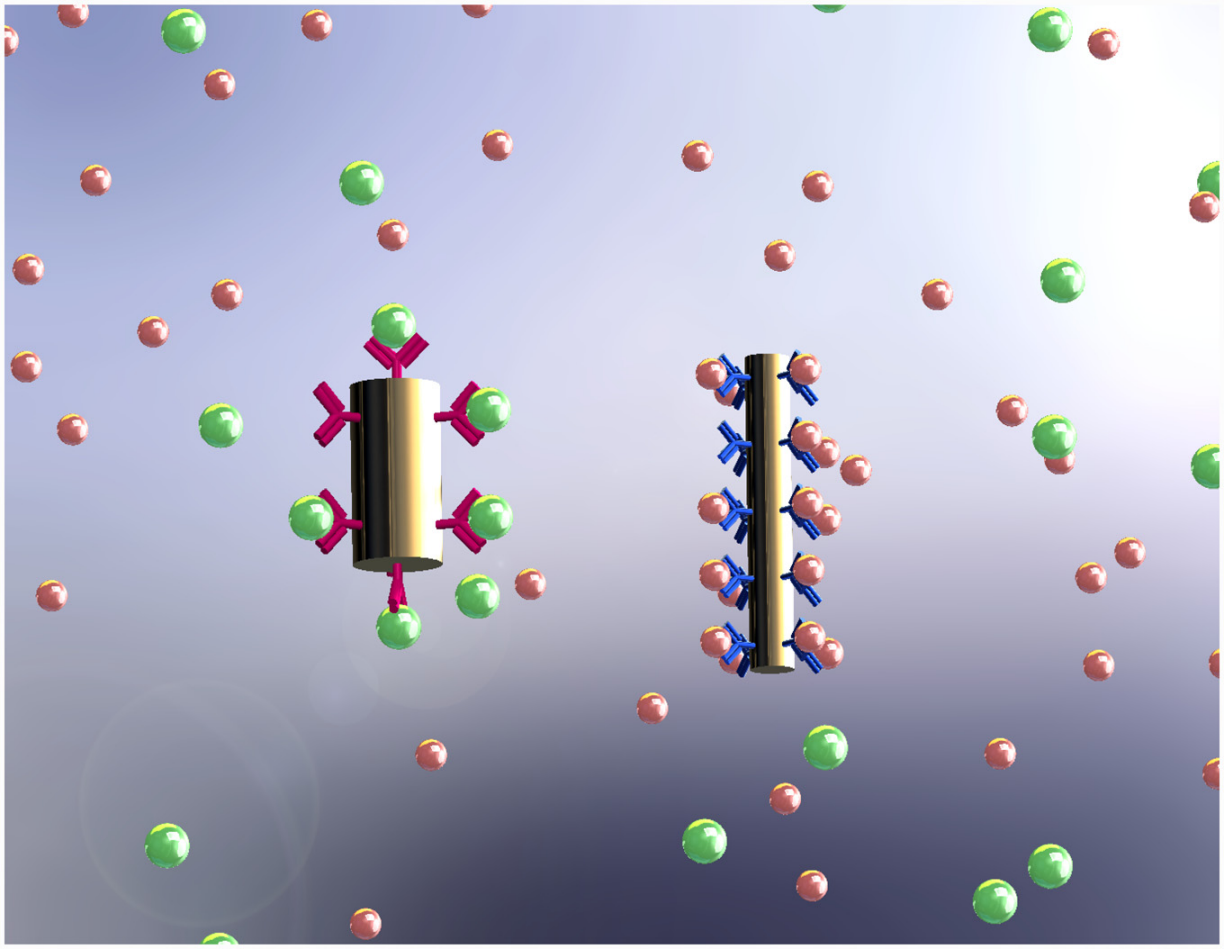
Two different nanorods functionalized with different receptors. Neurotransmitters selectively bind to the associated nanorods. Figure is drawn by E.F.

**Fig 2:**
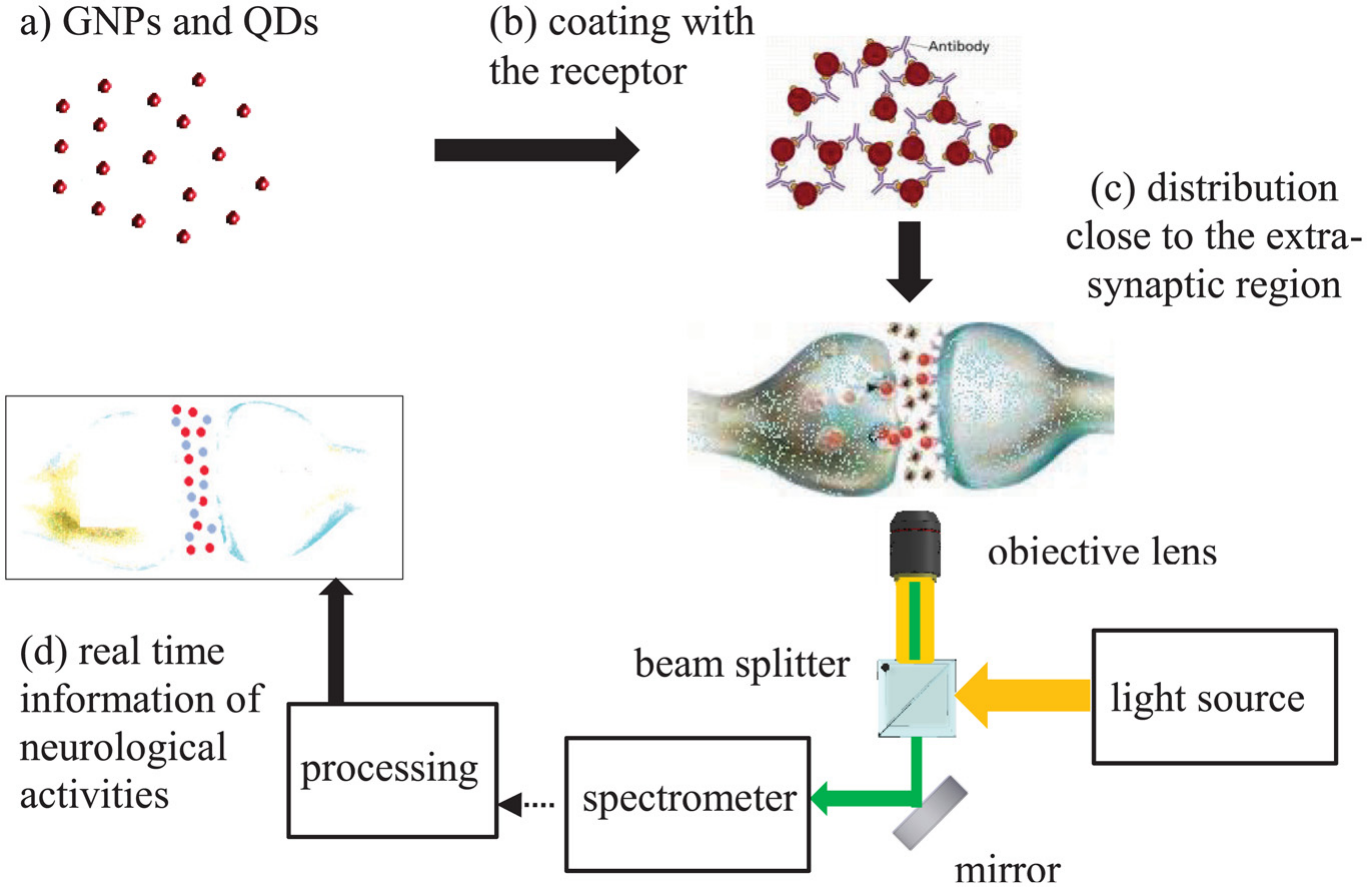
The proposed concept of optical real time imaging of neural activities using functionalized resonant nano-structures. Figure is drawn by A.S.

The idea in this work is to develop a technique whereby extra-synaptic dopamine (and other types of neurotransmitters) can be detected with cellular level resolution based on the optical properties of plasmonic nanoparticels, i.e. Localized Surface Plasmon Resonance (LSPR), or QDs. One advantage of this approach is that multiple neurotransmitters can potentially be identified simultaneously by using different nanoparticles. Resonant nanoparticles and QDs in this approach are functionalized with the appropriate receptors, as shown in Fig 1. This paper is specifically focused on developing solution-based dopamine sensors. As Fig 2 depicts the proposed idea schematically, functionalized nanoparticles are to be distributed over the neurons and within the synapses. During neural activities, the sparsely released dopamine in the extra-synaptic region of neurons will bind to the functionalized nanoparticles and will be detected optically by means of spectroscopy (reflection or photoluminescence). It is expected that the binding of neurotransmitters to the nanoparticles would change the optically reflected/transmitted light that can be detected effectively. In the following, we first study GNPs as the neurotransmitter’s detectors. Then, in the last section, we consider QDs and compare them with GNPs.

Surface Plasmons (SPs) are the collective charge oscillations at the surface of plasmonic materials. SPs coupled with photons form the composite quasi-particles known as Surface Plasmon Polaritons (SPPs) [16–18]. Theoretically, by solving Maxwell’s equations on both sides of a surface, it is easy to show that 3D materials with negative bulk permittivities (e.g., noble metals) or 2D materials with non-zero imaginary surface conductivities (e.g., graphene [19]) are essential in order to support the SPP. In addition, if the size of the 3D plasmonic material is limited (i.e. it forms a particle), the geometrical resonance of the particle will combine with the SPP to form LSPR. Unlike SPR, the excitation of LSPR can be easily done by plane waves which makes them suitable for practical applications [20, 21]. In other words, under illumination with a broadband source, plasmonic particles generate high intensity localized electric fields bound to their surface, making them good candidates for label-free bio-sensing applications [22, 23]. The shape, size, and material of the particle determine the localized electric field properties such as the intensity, the frequency, and the spatial distribution. Due to the inherent loss in plasmonic materials, and the loss in the material surrounding the particles, portions of the excitation wave will be absorbed and converted to heat in and around the surface of the nanoparticles. Besides being used in heat ablation therapy techniques [24–26], the absorbed energy can be used for sensing applications, simply by monitoring its absorption frequency spectrum [27–29]. At frequencies closer to the particle’s LSPR, the localized electric field and therefore the energy absorption (conversion to heat) is stronger. That is, any changes in the dielectric properties of the material in the immediate vicinity of nanoparticles will be reflected in the the absorption spectrum of the mixture either as a shift and/or as a change in its curvature. The sensitivity of the absorption spectrum of nanoparticles to the dielectric properties of their immediate vicinity is proportional to their localized electric field intensity which is in turn limited by their resonance quality factor defined as [29]

$$Q=\frac{E_{Local}}{E_{incident}}$$ (1)

in which *E*_*local*_ and *E*_*incident*_ are the intensity of the localized and the incident electric fields, respectively [30–32]. Among different noble metals, silver provides the highest electric filed concentration, as it has the lowest loss and can provide the highest resonant quality factor. However, silver nanoparticles have practical issues such as fast oxidization. The next suitable noble metal is gold, which is the most common material in LSPR based sensors. Gold has higher loss compared to silver, but has less practical limitations. Finding the resonance quality factor of plasmonic nanoparticles can be done using the classical computational (and sometimes analytical) electromagnetic techniques. As a known result, the quality factor of spheroids (and rods) increases as their aspect ratio increases [33, 34]. That is, prolate spheroids have larger quality factor than spheres and oblate spheroids.

## Methodology (GNPs)

**Fig 3:**
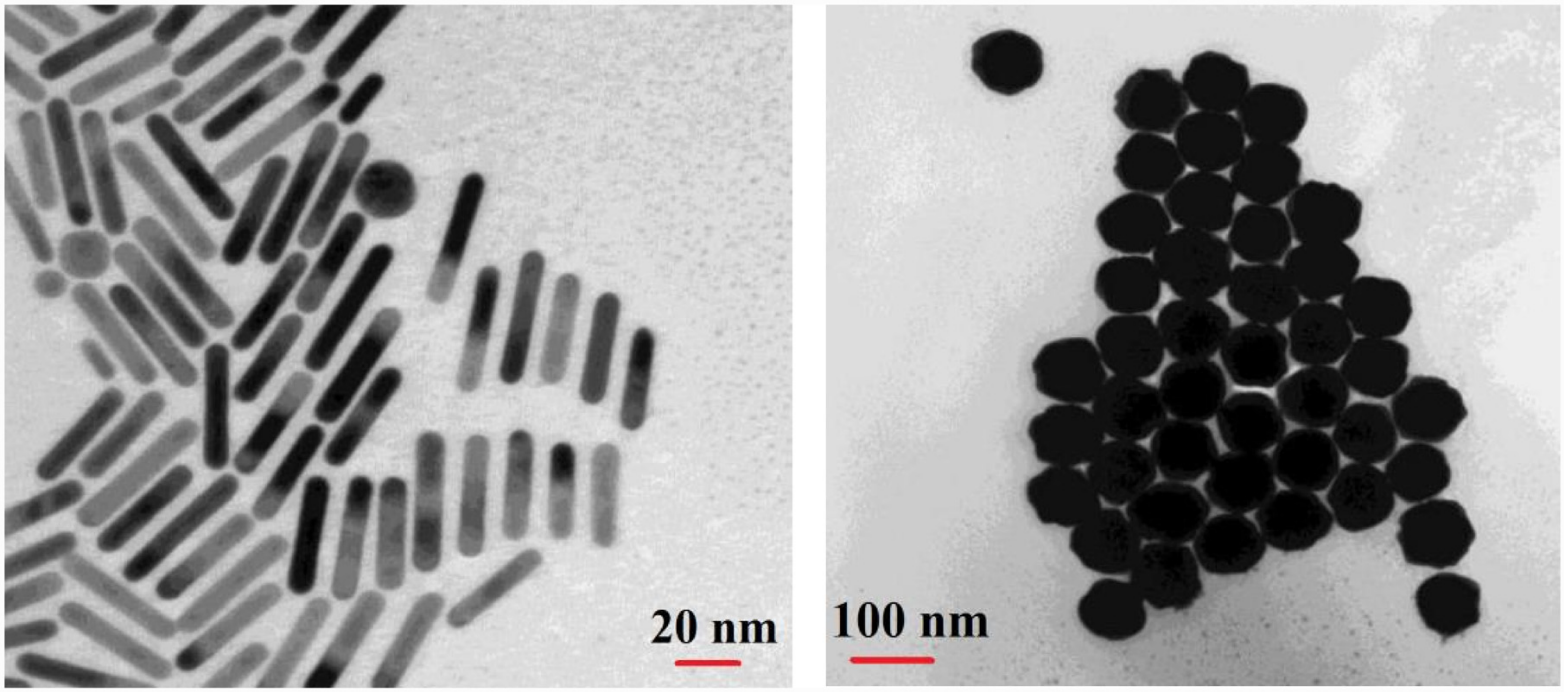
Example TEM images of the studies GNPs: NR6 (left), and NS5 (right) as introduced in Table 1.

Several gold nanoparticles were functionalized using different neurotransmitter receptors, and their absorption spectra are reported pre and post binding of neurotransmitters. We used commercially available GNPs (both spheres and rods) in order to keep the experiments reproducible for future investigations without concerns about the variabilities in nanoparticles synthesis. Table 1 lists the nanoparticles used along with their basic characteristics and Table 2 contains the different neurotransmitters and receptors which were used in this study. Sample TEM images of the studied GNPs are shown in Fig 3 as well.

**Table 1:** The studied GNPs (from Nanopartz Inc.).

Type	Label	Diameter [*nm*]	Aspect ratio	First abs. peak [*nm*]	Second abs. peak [*nm*]	Part no.
Rod	NR3	25	2.9	518	689	C12-25-700
Rod	NR5	25	2.4	517	662	C12-25-650
Rod	NR6	10	5.9	511	946	C12-10-980
Sphere	NS5	80	1	546	-	C11-80
Sphere	NS6	125	1	606	-	C11-125

**Table 2:** List of the studied Neurotransmitters and receptors.

Name	Type	Label	Provider	Part no.
Dopamine	Neurotransmitter	Dop	Sigma-Aldrich	H8502
GABA	Neurotransmitter	Gab	Sigma-Aldrich	A2129
Glycine	Neurotransmitter	Gly	Sigma-Aldrich	G7126
Anti-GABA antibody	Receptor	Antigab	Sigma-Aldrich	A2052
Anti-Dopamine antibody	Receptor	Antidop	abcam	ab6427
Anti-Glycine antibody	Receptor	Antigly	abcam	ab9442

To standardize the method, we used antibody against dopamine (Antidop) and cleaned it off using abcam anitbody cleaning kit. It is to be noted that Antidop would act as a receptor for dopamine released at the synaptic cleft. After determining the concentration, we used 3*μg*/*ml* concentration of antibodies to conjugate it to nanospheres. We left it overnight in cold room with gentle shaking for the antibodies to conjugate and then washed prior conjugating it with dopamine. The dopamine was freshly constituted in PBS and 100 micromoles were added to the antibodies bound to nanospheres accompanied by gentle shaking. The mixture was let to stand for 45 minutes followed intermittent gentle shaking. Carbonyl groups at the C-terminal of the Antidop interacts with the free amine groups of the nanopheres through electrostatic interaction. In spherical GNPs, the antibody cysteine could bind to the Au displacing the adsorbed citrate or other carboxylic acids used as capping agents. However, nanorods were capped by a Hexadecyltrimethylammonium bromide (CTAB) micelle which does lend itself to antibody cysteine binding. Therefore, the CTAB was replaced with a 12-Mercaptododecanoic acid NHS ester added at 1*μg* per 50 OD-mL of nanorods. After purification via centrifugation, 100 micrograms of Antidop were added and the mixture vortexed for 30 minutes. The NHS binds to the primary amines of the Antidop within 10–15 minutes. Three more purifications via centrifugation were used. All conjugations (spheres and nanorods) were done at 25 C and pH 7.4. Then, the sample was transferred into the well of 96 well plate and the absorption spectrum is recorded using Tecan infinite m200 plate reader.

We defined our standard results set as the comparison among three measured absorption spectra for each GNP: 1) bare GNP, 2) functionalized GNP with the receptor, and 3) neurotransmitter bound to the functionalized GNP. All of the absorption spectra were normalized to their maxima as we only relied on the frequency shifts. In each standard results set, the frequency shift (often redshift) in the GNP’s absorption spectrum after functionalizing ensured the proper attachment of the receptor.

## Results and Discussion (GNPs)

**Fig 4:**
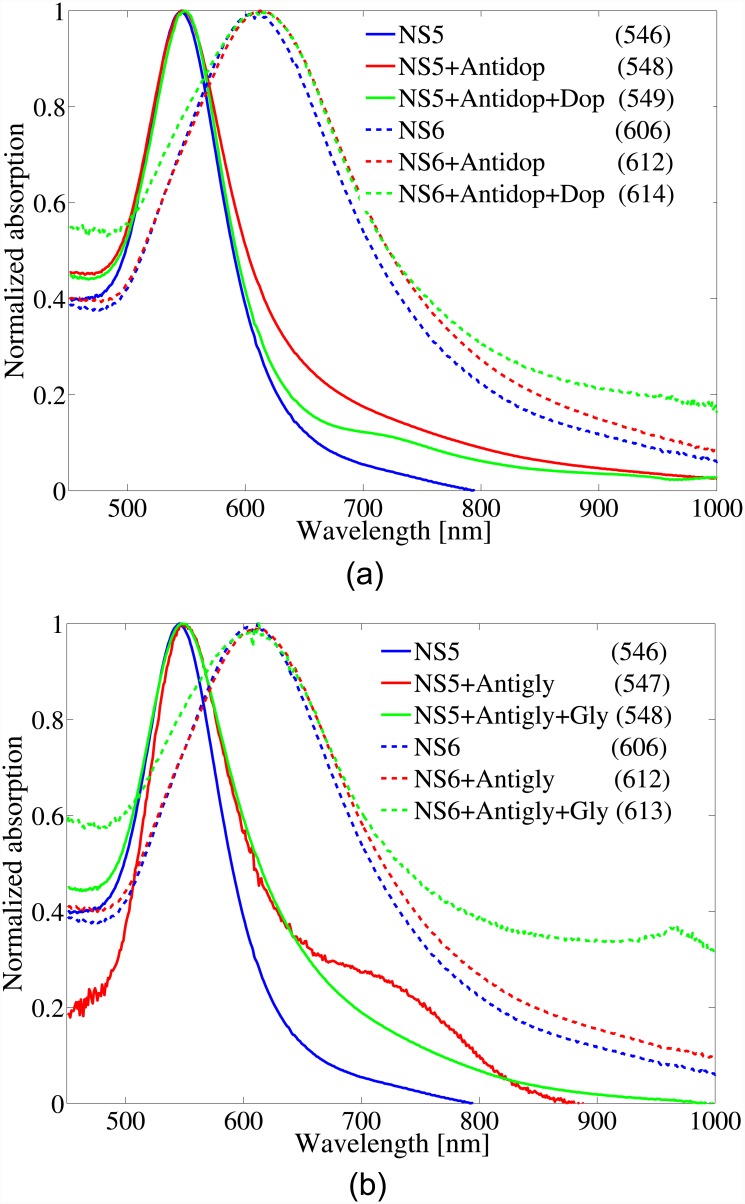
The standard results set for NS5 and NS6 nanospheres using a) Antidop as the receptor and Dop as the neurotransmitter, and b) Antigab as the receptor and Gab as the neurotransmitter. The numbers in parenthesizes report the peak wavelength of each curve in units of *nm*.

Fig 4(a) shows the standard results set for NS5 and NS6 with Antidop and Dop as the receptor and the neurotransmitter, respectively. It is evident that both nanospheres’ spectra showed detectable redshifts after adding Dop to the functionalized nanospheres. Moreover, as was expected, the larger nanosphere exhibited larger redshifts due to the larger surface area. Fig 4(b) also reports the standard results set for NS5 and NS6 but using Antigly and Gly as the receptor and the analyte, respectively, which showed redshifts 1*nm* post binding of the analyte. As a verification, the same result was repeated using the same size nanosphere from a different company (Innova Bioscience Inc.) and similar results were observed. It is worth mentioning that, due to the extreme confinement of the localized electric fields of LSPRs to the GNP’s surface, GNPs could only interact with the neurotransmitters if they were bound to each other.

**Fig 5:**
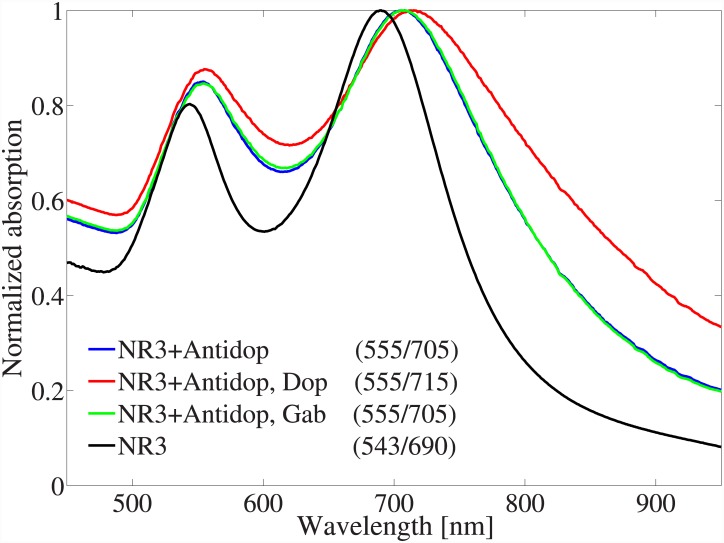
The standard results set for NR3 using Antidop as the receptor and Dop as the neurotransmitter. The numbers in parenthesizes report the peak wavelengths of each curve in units of *nm*.

After repeating the same experiments with smaller nanospheres than 80*nm*, we concluded that the shifts are too small to be detectable. The redshift in nanospheres with sizes larger than 80*nm* were repeatable and therefore proved the proposed concept. However, the sensitivity was still too small (2*nm* for NS6) to be useful in practical instrument designs. Besides increasing their surface area, the sensitivity of GNPs to neurotransmitters can be enhanced by choosing GNPs with higher resonance quality factor which in turn increases the localized electric fields intensities on the GNPs’ surface. Fig 5 reveals the standard results set for NR3 using Antidop and Dop as the receptor and the neurotransmitter, respectively. A redshift of 10*nm* post binding of Dop to the functionalized nanorod was observable in the second absorption peak in Fig 5. The enhanced shift in the spectrum of the nanorod compared to nanospheres is due to its higher resonance quality factor which in turn leads to a higher localized electric field based on Eq (1). Equivalently, by forming and solving an electromagnetic scattering problem, it is straight forward to show that prolate spheroids (which can be approximated by nanorods) have higher absorption cross section than spheres and oblate spheroids [34]. In fact, in the extreme limits of a spheroid, the absorption cross section of a lossy needle is several orders of magnitude larger than that of a disk (see Fig 2 in [34] which applied to any lossy spheroid on a different scale). Higher light absorption means stronger interaction with light, larger localized electric fields around the particle, and higher sensitivity to the environment.

The first and the second absorption peaks in nanorods’ absorption spectra were associated with transverse and longitudinal resonance modes, respectively. As a control, Gab was also added to the NR3 functionalized with Antidop which did not have any noticeable effect on its spectrum. Gab (GABA) is also a neurotransmitter which acts on inhibitory synapse.

**Fig 6:**
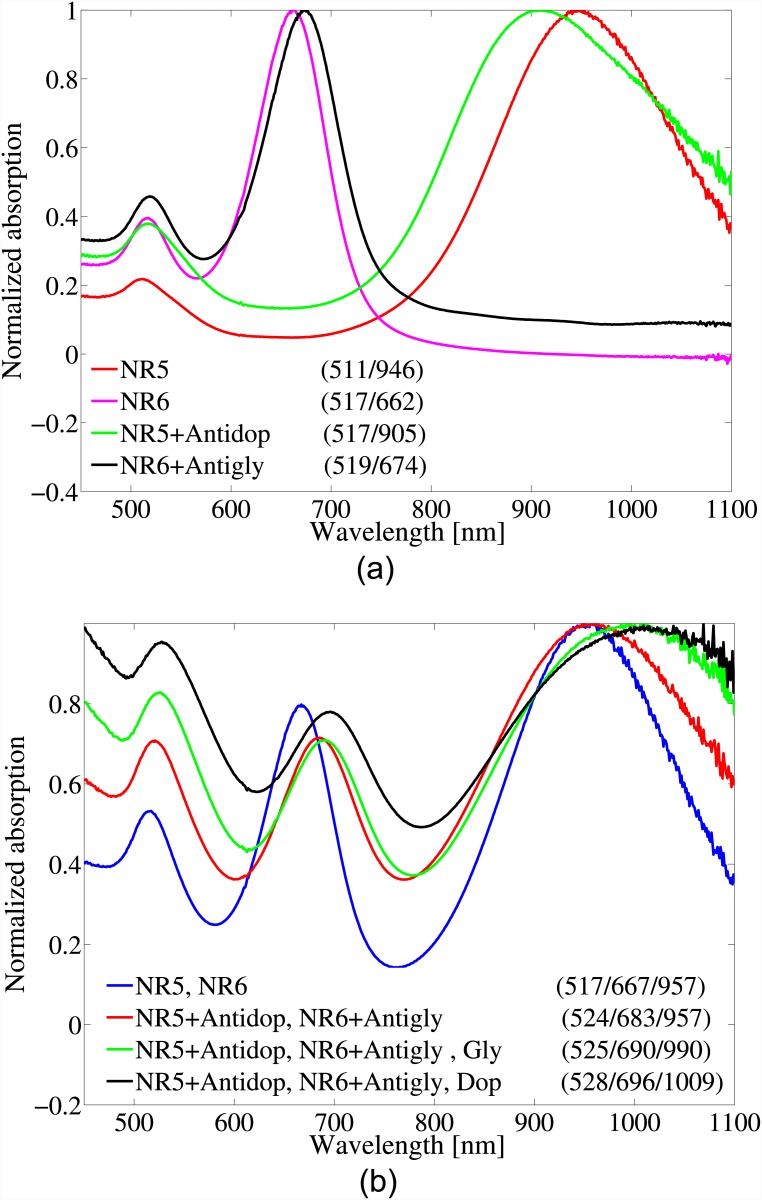
a) Absorption spectra of NR5 and NR6 before and after functionalizing with the receptors, and b) using the mixture of the functionalized GNPs to detect both Dop and Gab. The numbers in parenthesizes report the peak wavelengths of each curve in units of *nm*.

As a further investigation, NR5 and NR6 were functionalized with Antidop and Antigly, respectively. The two functionalized GNPs were then mixed together and Gly or Dop was added to the mixture consequently. The idea in this measurement set was to examine the possibility of discerning neurotransmitters using a mixture GNPs with different sizes so that each size was functionalized with a specific receptor. Fig 6(a) shows the spectra of NR5 and NR6 before and after binding to the receptors. The shift in the peak wavelength confirmed proper GNP functionalizations. Note that unlike the nanorods with diameter 25*nm* (and nanospheres), the longitudinal resonance of the nanorods with diameter 10*nm* experienced a blue-shift post binding of Antidop (this was also observed in measurements using different nanorods). On the other hand, after mixing the two functionalized GNPs, both of the longitudinal peaks experienced a larger redshift (canceling the blue shift of the second peak), which could be due to the charge effects in the solution. Fig 6(b) includes the spectrum of the mixture of the bare nanorods with two longitudinal resonance peaks at 667*nm* and 957*nm* which the first peak moved to 683*nm* post binding of antibodies. The first and the second peaks redshifted 7*nm* and 33*nm*, respectively, post binding of Gly which were different form the 13*nm* and the 52*nm* redshifts post binding of Dop.

## Replacing GNPs with QDs

**Fig 7:**
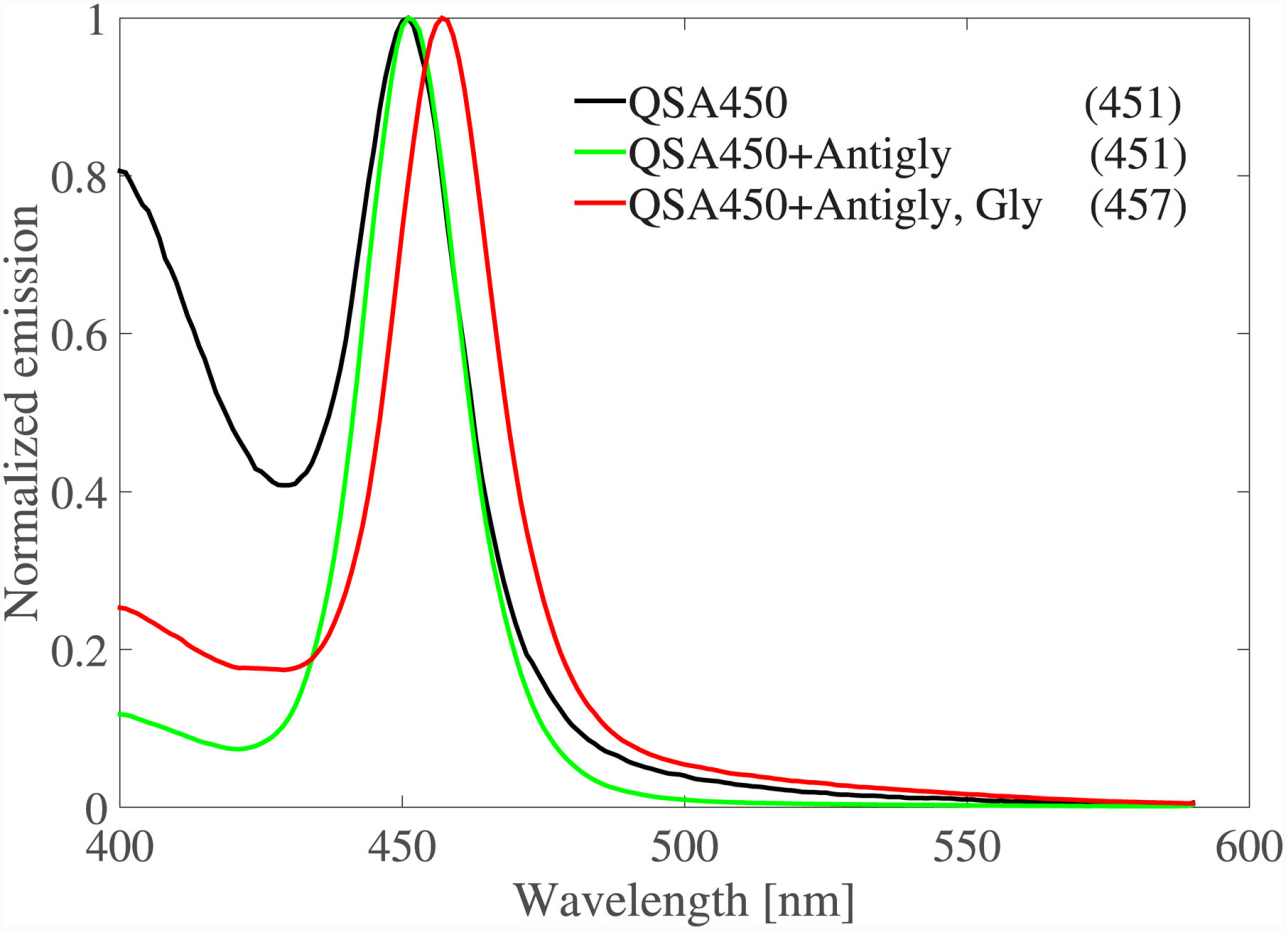
a) The standard results set for QSA-450 using Antigab as the receptor and Gab as the neurotransmitter. The pump wavelength was 300*nm* and the numbers in parenthesizes report the peak wavelength of each curve in units of *nm*.

**Fig 8:**
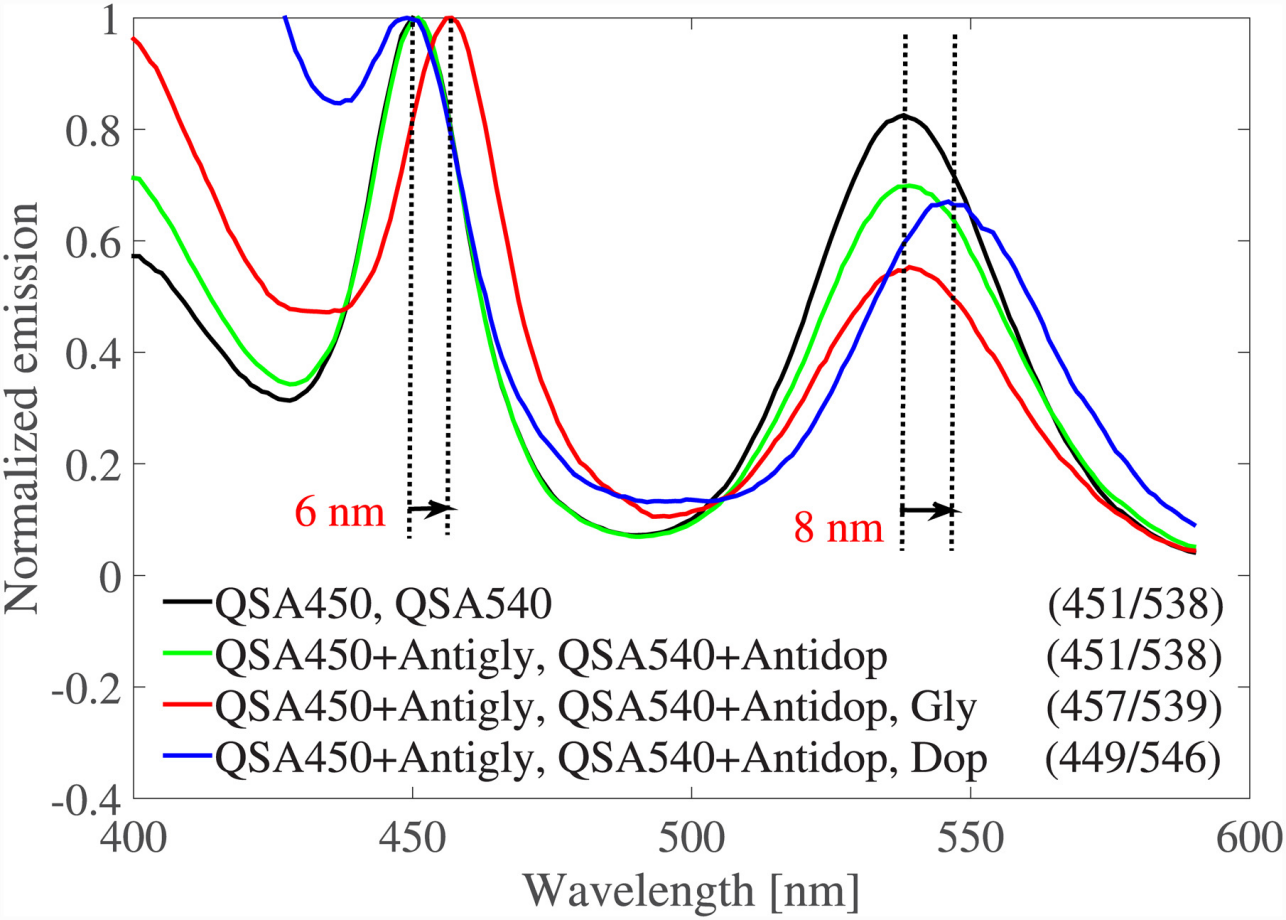
a) Emission spectra of the mixture of QSA-450 and QSA-540 functionalized with Antigab and Antidop, respectively. Dop and Gab are added to the mixture separately. The pump wavelength was 300*nm* and the numbers in parenthesizes report the peak wavelengths of each curve in units of *nm*.

The absorption spectra of gold nanorods showed around 10*nm* redshift after binding to neurotransmitters. However, the peak in GNPs spectra is wide, due to the loss, which is not desirable in detecting the peak shift. In other words, the ratio of the peak shift (after binding to the analyte) to the peak width is a better measure of detection convenience in our approach. Therefore, nanoparticles with much higher resonance quality factor are preferred in this method. Alternatively, instead of GNPs, we can use QDs which typically have very narrow emission spectra. Quantum dots are two/multi level quantum systems which can emit photons with energies equal or greater than their bandgap, if pumped with high enough energy photons. The bandgap of a QD, as well as its excited and ground levels quantization, are mainly controlled by its size and material. However, particles in the vicinity of QD, along with the ambient medium, impose extra restrictions on the emitted spectrum, which is often explained via the Purcell effect [35]. In [36, 37], effects of plasmonic materials (GNPs and graphene) on the emission spectrum of a quantum dot are studied theoretically by forming and solving a quantum master equation [36, 38]. We studied two commercially available CdSSe/ZnS core/shell QDs from Ocean NanoTech [39] with emission peaks at 450*nm* (part no. QSA-450) and 540*nm* (part no. QSA-540). The full width of half maximum (FWHM) of the emission spectra of the studied QDs were 35*nm*. QDs were sourced from Ocean NanoTech, and were amine capped. 1:1 Molar ratio of active amine sites were activated with Thermo Fisher BS3 and then conjugated to the antibody. BS3 (Sulfo-DSS) is bis(sulfosuccinimidyl)suberate, an amine-to-amine crosslinker that is homobifunctional and water soluble. Excess 5:1 antibody was added and then purification was performed by dialysis. It was determined that extra amine groups were not readily purified when the QDs were received. Therefore, a pre-purification using dialysis was completed. all conjugations were performed at 25 C and pH 7.4 in PBS. QDs were pumped at 300*nm* wavelength and their emission spectra were measured using a florescence spectrophotometer. Fig 7 shows the standard results set for QSA-450 using Antigly as the receptor and Gly as the neurotransmitter. The emission peak of QSA-450 was shifted 6*nm* after binding to the neurotransmitter. The ratio of the peak shift to the FWHM for QSA-450 is 0.18, which is twice as the 0.09 ratio for NR6. This makes QD a definitely better choice than GNP to be used in our approach. Moreover, the excitation and emission wavelengths are different in QDs (the excitation is at a smaller wavelength, 300*nm* in our experiments) which makes emission detection much easier. This is a great advantage over GNPs whose scattering is at the same wavelength and much weaker than the excitation. Similar to GNPs, mixture of different QDs coated with different receptors can be used to discern neurotransmitters. Fig 8 includes the emission spectrum of the mixture of QSA-450 and QSA-540 with peaks at 451*nm* and 538*nm*. Then, QSA-450 and QSA-540 were coated with Antigly and Antidop, respectively. The emission spectra of the functionalized mixture before and after adding Gly and Dop are shown in Fig 8. The first peak in the spectrum of the functionalized mixture shifts 6*nm* post binding of Gly while the second peak remains almost at the same wavelength (with the tolerence of 1*nm*). Likewise, the second peak shifts 8*nm* post biding of Dop. This is exactly what we intended, as Dop(Gly) can only attach to and interact with QSA-540(QSA-450). Therefore, only the peak associated with QSA-540(QSA-450) changes in the mixture emission spectrum. Dop(Gly) cannot interact with QSA-540(QSA-450) because their distance is too large (since they are not brought together by receptors).

In summary, this paper provides a good solid lead for functional brain mapping using GNPs and QDs for dopamine, glycine and GABA sensing neurons. The large frequency shifts in the spectrum of the mixture of nanorods, as well as its specificity to different neurotransmitters, confirmed the proposed functional brain mapping techniques. However, the broad absorption peak of GNPs is an obstacle in detecting the peak shift. Finding a set of nanoparticles with much higher quality factor (e.g. dimers) and a range of resonance frequencies can ensure practicality of using GNPs. As another approach, in order to improve detection, QDs were used instead of GNPs. Since the emission spectrum of a QD is narrow, its peak shift to its FWHP ratio is large, which helps the detection of neurotransmitters. Moreover, the excitation wavelength of QDs is different than their emission spectra, which benefits the detection further. Whether these GNPs and QDs are toxic to the cells and tissues is yet to be confirmed. It will be also important to test the sensitivity of these GNPs and QDs once they penetrate the tissues.
